# Supplementary material for: Visual crowding is a combination of an increase of positional uncertainty, source confusion, and featural averaging
Source: Sci Rep. 2017 Apr 5;7:45551. doi: 10.1038/srep45551 (PMC5381224; doi:10.1038/srep45551)
Supplement: Supplementary Results [file srep45551-s1.pdf]

Visual crowding is a combination of an increase of positional uncertainty, source confusion, and featural averaging

William J Harrison [1,2] & Peter J Bex [3]

[1] Department of Psychology, University of Cambridge; [2] Queensland Brain Institute, The University of Queensland; [3] Department of Psychology, Northeastern University

Appendix A – Supplementary Analyses

Prior to the mixed-model analyses for the one-gap and two-gap flanker conditions presented in the Results section, we performed the following analyses.

One-gap flanker condition

Shown in Figure A1A are the naïve observer's report errors and linear fits as a function of target-flanker orientation difference. Note that a linear fit with a slope of 1 would indicate the observer's reports followed the flanker gap closely, a slope of 0 would indicate the observer's reports were not influenced by the flanker, and a slope of 0.5 conforms to the average of target and flanker orientations. In Figure A1B-D we plot the slope parameter for all conditions from all observers for the one-gap flanker condition. Slope parameters were close to 0.5 for the two smallest flanker sizes, and generally decreased as the flanker size increased. For all observers, the slopes were approximately 0 for the largest flanker condition. However, these analyses do not describe the data in full (see Results).

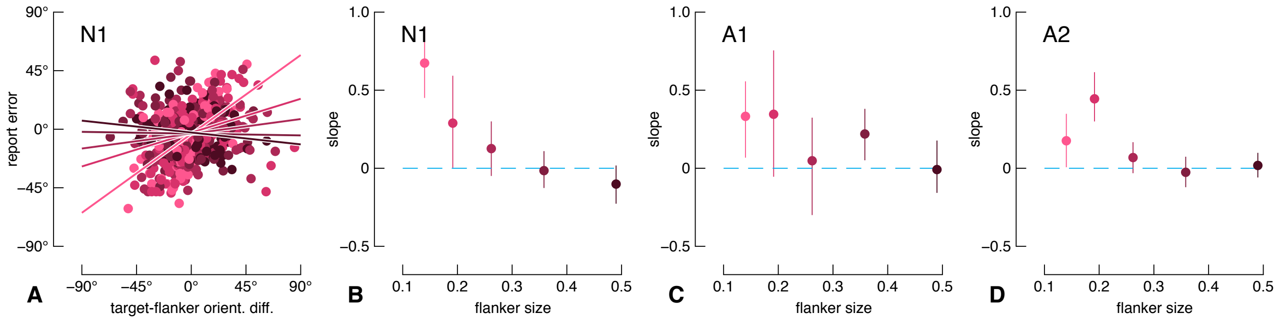

Figure A1. Results from linear fits in the one-gap flanker condition. A) Example raw data from the naïve participant. Lines show linear fits for each target-flanker separation, with colour shading indicating conditions as in Fig. 2A. To improve visibility, we have truncated the x-axis and y-axis. B-D) Slope parameters as a function of flanker size for all observers. Errors bars are 95% confidence intervals.

Alternative analyses

Shown in Figure A2 are results from further alternative analyses (see Alternative analyses section of the Methods). In brief, these analyses assume reports consist of only target and substitution errors, rather than also including average errors as in the main Results. The mixture modelling was equivalent to the "swap" model used by Bays et al [1], and applied using the MemToolbox [2]. It provides an estimate of the proportion of substitution reports, as well as the perceptual error, which we show in Figure A3 in comparison with error for the no-gap flanker condition. This analysis also estimates the proportion of random guesses: this parameter was never more than 7% for any observer or condition, but was often 0, consistent with infrequent lapses. The Monte Carlo analysis in Figure A2 involved two main steps, but we provide more detail below. First, we used ROC analyses to quantify the proportion of report errors accounted for by each model as a function of the distance from the model slope, with residual distances ranging from 0° to 45°. Second, we then calculated the area under the ROC (AUC) as a measure of sensitivity of each model. Across two thousand simulations, we randomly labelled a subset of data as belonging to the substitution model, while the rest of the data were attributed to the target model, repeating the AUC analysis on each run. The proportion of substitution reports was taken as the proportion that maximized the sum of the two AUCs. The results of these analysis show the high proportion of substitution errors, consistent with our main analyses.

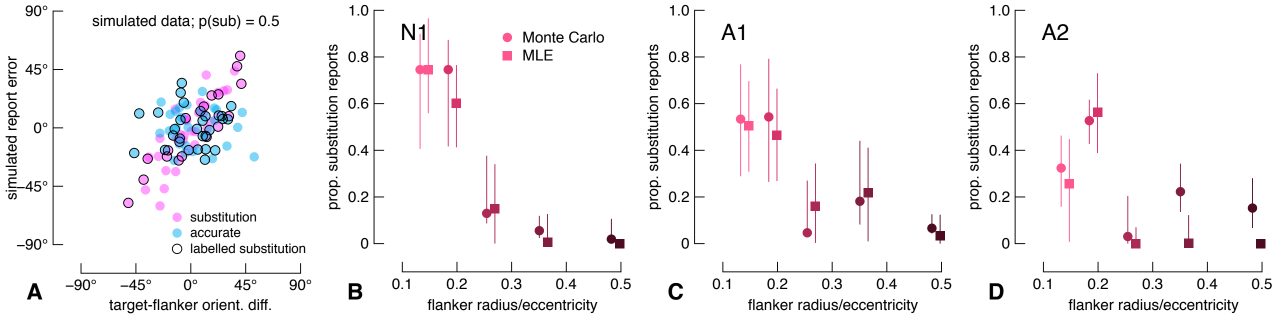

Figure A2. Results from Monte Carlo and mixture modelling of the one-gap flanker condition. A) Example of Monte Carlo analysis. Simulated data are a mix of accurate and substitution errors. Each datum had 0.5 probability of being labelled a substitution; across many iterations, such probabilistic labelling provides accurate estimates of underlying error proportions (see below text for details). B-D) Estimates of proportions of substitution reports for all observers using MLE (mixture modelling) and Monte Carlo analysis. Error bars are 95% bootstrapped confidence intervals.

We explain the steps of the Monte Carlo analysis in further detail using a simulated dataset in which we know the proportion of substitution reports. The data shown in Figure A2A are 100 simulated observations; 50% of the data are accurate reports (i.e. generated by the target model; blue data) and 50% are generated by the substitution model (pink data) with added Gaussian noise to approximate observers' report variability (s.d. = 15°; see Fig. 2). In a single Monte Carlo simulation, we probabilistically assigned each datum to the substitution model. In Figure A2A, the probability of each observation being classed as substitution was 0.5, and data classed as substitutions are indicated by black circles. We then calculated the AUC for both target and substitution models as described above, but with mutually exclusive datasets as determined in the previous step. We found the sensitivity of the combined models by simply summing the target AUC and substitution AUC, giving a "mixed AUC". Note that, in Figure A2A, both the proportion of substitution data and the probability of classifying each observation as a substitution was 0.5, but, due to the probabilistic nature of classification, some observations were mislabelled, which results in a relatively low mixed AUC for this specific simulation. We thus perform 50 such simulations, increasing the probability that a closer-to-optimal set of classifications will occur at least once, which we infer by the maximum AUC from all simulations at a given probability of substitution. For each condition and each observer's dataset, we repeat this procedure for all probabilities of a substitution, from 0 to 1 in steps of 0.01. The resulting mixed AUC values across proportions of substitutions were approximately Gaussian. To determine the proportion of substitutions yielding the highest AUC, we thus took the mean of a Gaussian fit to these data. This method yields accurate estimates for data with known model proportions. Variability of each estimate was found by bootstrapping each dataset 400 times and repeating the above process for each bootstrapped dataset, giving a total of 20000 (50 x 400) simulations. The numbers of simulations and bootstraps were determined by ensuring estimate accuracy with known data while remaining computationally tractable.

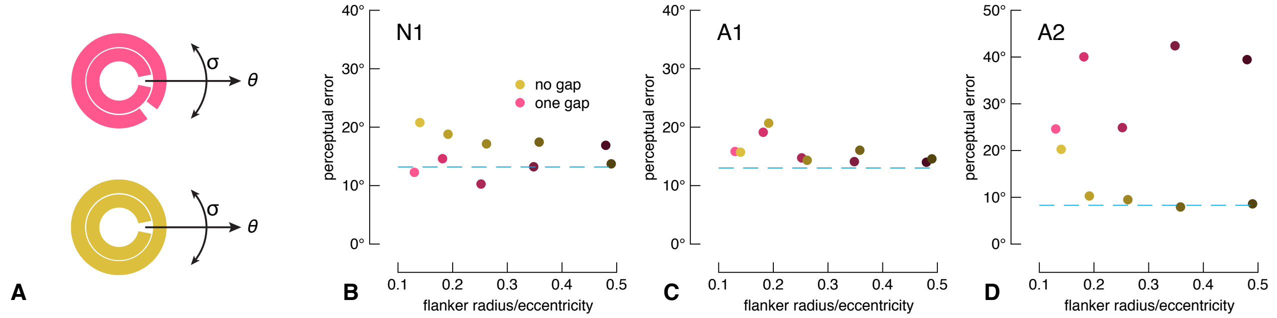

Figure A3. Variability of report errors across flanker types. A) We compared the variability of target-centered reports for the one-gap (top) and no-gap (bottom) flanker conditions. B-D) Perceptual error plotted as a function of flanker size for each participant. For the no gap condition, perceptual error is the circular standard deviation of report errors (replotted from Fig. 2), while it is the same parameter estimated by MLE for the one gap condition. The dashed line indicates perceptual error for the unflanked condition. Note the scale of the y-axis is different for A2 compared with the other observers.

Two-gap flanker condition (near-gap)

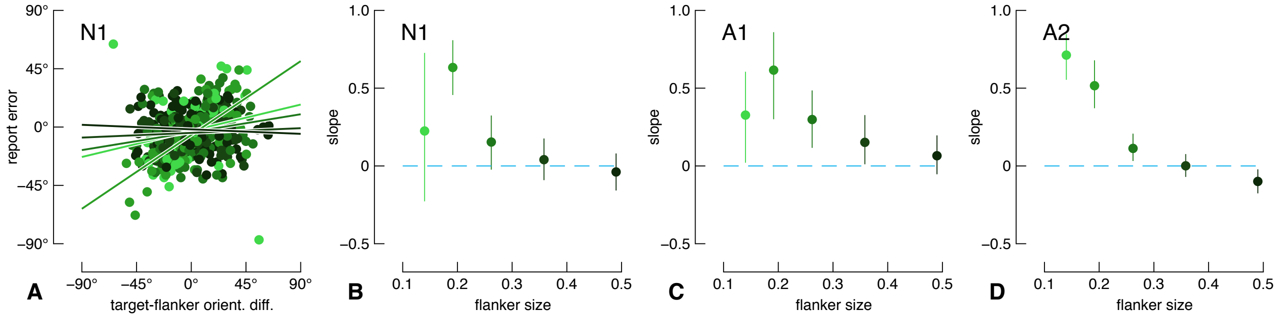

Figure A4. Results from the two-gap flanker condition for report errors less than 90° from the target. A) Raw data from the naïve participant. The x-axis indicates the orientation difference between the target gap and the nearest flanker gap. Data presented as in Fig. A1A. B-D) Slope parameters as a function of flanker size for all observers. Errors bars are 95% confidence intervals.

Supplemental References

1. Bays, P. M., Catalao, R. F. G. & Husain, M. The precision of visual working memory is set by allocation of a shared resource. *Journal of Vision* **9,** 7.1–11 (2009).
2. Suchow, J. W., Brady, T. F., Fougnie, D. & Alvarez, G. A. Modeling visual working memory with the MemToolbox. *Journal of Vision* **13,** (2013).
